# 801 Topical hemostatic agents in burn surgery: a systematic review

**DOI:** 10.1093/jbcr/irac012.350

**Published:** 2022-03-23

**Authors:** Andrea Battistini, Sebastian Q Vrouwe

**Affiliations:** University of Chicago, Chicago, Illinois; University of Chicago, Chicago, Illinois

## Abstract

**Introduction:**

Acute burn surgery (tangential excision and grafting) has long been associated with significant intra-operative bleeding over large surface areas, where in adults approximately 200-250 mL of blood loss would be expected per %TBSA excised and grafted using traditional methods. Several techniques were introduced to limit bleeding, including tourniquets, tumescent infiltration, and topical agents. To date, no study has comprehensively investigated the available data regarding topical hemostatic agents in burn surgery.

**Methods:**

A systematic review was performed by two independent reviewers using PubMed, Scopus, and Web of Science databases from first available to September 10, 2021. Articles were included if they were published in English and described or evaluated topical hemostatic agents used in acute burn surgery (excision and/or grafting). Animal studies and review articles were excluded. Data was extracted on the topical agent(s) used, their dosage, mode of delivery, hemostasis outcomes (if measured), and complications (if reported).

**Results:**

The search identified 1982 non-duplicate citations, of which 134 underwent full text review, and 49 met inclusion criteria. Papers were grouped whether they compared (n=11, 22%), described (n=21, 43%) or secondarily described (n=17, 35%) topical hemostatic agents. Several authors (n=22, 45%) described topical hemostatics as part of a protocol that included other methods of blood conservation (tourniquet, tumescent infiltration, etc). In total, 31 studies incorporated a vasoconstrictor agent (epinephrine, phenylephrine, vasopressin), and 30 studies incorporated a procoagulant agent (thrombin, fibrin). Four studies incorporated other agents (hydrogen peroxide, tranexamic acid and collagen). The most common vasoconstrictor used was epinephrine, with doses ranging from 1:1,000 to 1:1,000,000. The most common procoagulant used was thrombin, with doses ranging from 10 to 1000 IU/mL. Among the comparative studies, outcomes of blood loss were not reported in a consistent manner, therefore meta-analysis could not be performed. The majority of studies (94%) were level of evidence III to V.

**Conclusions:**

A multitude of topical hemostatic agents have been reported in the burn literature, with a wide range of dosages and modes of deliveries, as well as protocolization with other blood conservation techniques to limit blood loss during surgery. Determining the optimal topical hemostatic agent is limited by low quality data and challenges with consistent reporting of intra-operative blood loss and other clinically meaningful outcomes.

